# Microalbuminuria in type 1 diabetes mellitus children in University of Port Harcourt Teaching Hospital, Nigeria

**DOI:** 10.11604/pamj.2020.36.161.23782

**Published:** 2020-07-07

**Authors:** Iroro Enameguolo Yarhere, Tamunopriye Jaja, Mirabelle Anolue

**Affiliations:** 1 Endocrinology Unit, Paediatrics Department, University of Port Harcourt Teaching Hospital, Port Harcourt, Nigeria

**Keywords:** Diabetes, mixtard, basal bolus, microalbuminuria, justice

## Abstract

**Introduction:**

glycaemic control is usually best achieved using the basal bolus regimen, however, this is not always available in resource-limited settings. Long-term complications like renal parenchymal disease are consequences of poor glycaemic control. Screening type 1 diabetes patients irrespective of their disease duration was used to buttress the need for ethical principles of justice to be incorporated in the care of type 1 diabetes children.

**Methods:**

urine albumin creatinine ratio (UAC) was calculated for 20 type 1 diabetes mellitus children in the endocrinology clinic after submitting early morning urine over a 4-month period. The calculated ratio was compared between duration of disease (< 5 years and > 5 years) and between insulin regimen types (mixtard and basal bolus). Repeat tests were done for children who had elevated UAC ratio levels after 2 months.

**Results:**

there were 5 males and 15 females and the mean UAC ratio of the cohort was 123mg/g with a range of 5.30 − 906 mg/g. Twelve children (8 diagnosed less than 5 years) had UAC ratio ≥ 30mg/g with a mean of 193.15. The repeat mean UAC ratio for these was 144.35 mg/g. Children who had diabetes for more than 5 years and were on mixtard had higher UAC ratio than those with diabetes < 5 years and on basal bolus.

**Conclusion:**

the prevalence of microalbuminuria is high in our cohort of type 1 diabetes children and these were children on mixtard and had diabetes greater than 5 years.

## Introduction

The increasing prevalence of type 1 diabetes mellitus in children in the world and indeed Nigeria makes for improving the care and management of these children to prevent long term micro and macro-vascular complications [1-3]. The risk of these complications is increased with reduced diabetic control, high HbA1c levels and elevated blood pressure levels [2-4]. Generally, children are said to have better controls than adults as they are monitored by their parents and the EDIC trial shows that with basal bolus regimen, there is reduced risk for macro and micro vascular complications [5]. In Nigeria, every child with diabetes is self pay and not under any insurance so many receive mixed insulin or free mix insulin regimen while a few are on basal bolus regimen that requires insulin administration for every meal with a long acting at night or morning. This predisposes them to poor glycaemic control couple with their inability to regularly check blood glucose levels due to financial instability of most patients as with many other African countries like Tanzania, Rwanda [4, 6]. Though it is recommended to check for renal abnormality after 5 years with microalbuminuria [5, 7], it is our belief that children in Nigeria should be monitored with increased frequency due to perceived poor control and this prompted this present study. Until recently, many children being managed for diabetes were on mixtard insulin regimen, most of them receiving them for free following donation from Life for a child foundation like in Rwanda [6]. However, with increasing awareness and following reviews in guidelines, many children are now been counselled to commence the basal bolus insulin regimen. The aim of the study was to find the prevalence of microalbuminuria in children with type 1 diabetes mellitus in our clinic and if there was any difference between children on basal bolus regimen and those on mixtard, noting from previous studies like the EDIC that basal bolus insulin regimen helped in reducing long term complications of diabetes [8, 9].

## Methods

**Study setting:** all children diagnosed with and on follow up for type 1 diabetes mellitus in the University of Port Harcourt Teaching Hospital were recruited into the study after retrieving informed verbal consent from them and ascent from their parents, in the clinic from 1^st^of September 2018 to 30^th^of January 2019.

**Study design:** this is a cross sectional study of children with type 1 diabetes mellitus in a Teaching Hospital checking their risk for renal complications.

**Sample and data collection:** through telephone conversation the week and subsequently, a day before, instructions were given to fast for 8 - 10 hours before checking blood glucose in the morning and collecting first urine sample into a universal container and kept in ice before coming to the hospital. Urinalysis was done in all children looking for markers of urinary tract infections and there were no nitrites, protein, blood or leucocytes. The samples were immediately transported in an icebox to the chemical pathology research laboratory for analyses for urine albumin excretion, urine creatinine excretion, and the urine: albumin excretion ratio calculated. Albumin was measured from the urine sample using the immunoturbidity method with BSA 3000 machine Bordeaux, France and urine creatinine was measured using the kinetic method with the same BSA 3000 spectophotometry machine. The ratio was calculated using the formula urine albumin/ urine creatinine and this was converted to mg/g. The weight, height, hip and waist measurements were done using standardized protocols. The weight for age SDS, and height for age SDS were calculated using the CDC USA 2000 reference values. Other variables assessed were the socioeconomic status of parents using the Oyedeji classification, family history of diabetes mellitus, insulin dose, HbA1c within the last 3 months, average frequency of blood glucose check within the last 3 months, presence and type of complications and insulin regimen (basal bolus or mixtard). Those children with high urine albumin: creatinine ratios were recalled for repeat testing 2 months after the initial testing to confirm persistence of renal disease. They also had renal ultrasound scan done to check for renal parenchymal disease like loss of cortico-medullary differentiation and the sizes of the kidneys by a radiologist. They were counselled on need to either improve their glycaemic control for failure to do comply could lead to permanent kidney damage and end stage renal disease. **Statistical analysis:** the summary data were analysed using IBM SPSS 24, presented as charts and graphs and mainly describing means and SD of continuous variables, and proportions for categorical variables. Students t test was used to check differences in means between children on Basal bolus regimen and mixtard or free mix insulin regimen. Chi squared test was also used to test differences in categorical variables and for all statistical analyses, significance in difference was set at p <0.05.

## Results

Of the 22 children being managed in the clinic, 20 gave informed consent and were subsequently recruited into the study giving a compliance rate of 90.2%. There were 5 (25%) males and 15 (75%) females and the average age of children was 13.27 ± 3.68 years with a range of 5-18 years. The average duration of diabetes since diagnosis was 2.9 ± 0.44 years with a range of 1- 9 years and 3 children had a first-degree family history of diabetes mellitus but these were type 2 diabetes. Three children in low SEC on basal bolus started this regimen a year prior due to complications and attempt to reduce their HbA1c levels. The mean diastolic and systolic BP of the participants was normal with no differences between insulin regimen groups. However, when the population was separated according to duration of disease, those with duration within 5 years had significantly lower systolic BP, p = 0.020, but not the diastolic BP, p = 0.073. There was no significant difference in the mean systolic and diastolic BP of children with and without microalbuminuria, p = 0.906 and 0.394 respectively.

**Anthropometry and other clinical features:** the average weight for age SDS was 0.15 with a very wide range of -2.74 to 2.7 and the average height for age SDS was -0.29 with a wide range of -3.49 to 2.93. Two children had weight and height SDS less than - 2, and 2 were also greater than +2 SDS. The weight and height SDS were not different between children who had diabetes for <5 years and those with diabetes for > 5 years. Nine (45.0%) of the children were on basal bolus regimen and 11 (55.0%) were on mixtard in the past 1 year before the study commenced. Most of the children were in the middle socioeconomic class, 11(55%), and 7 (35%) were in the low socioeconomic class and this difference was significant p = 0.047. Fifteen (75.0%) children had diabetes for less than 5 years before the study was conducted and only 7 (43.7%) children tested their blood glucose at an average of 3 times per day or more.

**Microalbuminuria:** over the 5-month period, a total of 32 urine samples were collected from the 20 patients studied. Elevated urine albumin creatinine ratio was found in 12 (60%) patients who subsequently gave repeat samples within 3 months of the initial submissions. There was no age difference between those with elevated UAC ratio and those with normal levels 13.00 and 13. 44 years, p value = 0.801, Table 1. The median urine albumin: creatinine ratio in the population was higher than the cut off for microalbuminuria at 68.15 mg/g with a very wide range of 5.30 - 906.10 mg/g and mean of 123.69 mg/g.

**Table 1:** baseline characteristics of children according to the insulin regimen as at start of the study

Variable	Mixtard = 11	Basal bolus n = 9	All = 20	p value
**Mean age years (SD)**	14.9 (2.9)	11.2 (3.6)	13.2 (3.9)	0.02
**Duration (<5 years)**	9	6	15	0.43
**Sex of respondents F (%)**	10 (90.1)	5 (55.6)	15	
**Weight for age SDS (SD)**	0.17 (1.6)	0.13 (1.6)	0.15 (0.1)	0.96
**Height for age SDS (SD)**	-0.76 (1.6)	0.29 (1.7)	-0.29 (1.6)	0.17
**SBP mmHg (SD)**	100 (8.3)	95 (5.2)	98.5	0.11
**DBP mmHg (SD)**	68 (8.7)	65 (7.9)	66.8	0.40
**HbA1c % (SD)**	10.2 (4.3)	11.4 (3.4)	10.7(3.9)	0.50
**Urine albumin mg (SD)**	3.7 (3.0)	2.9 (1.4)	3.33 (2.4)	0.47
**Urine creatinine g (SD)**	6.0 (5.4)	5.1(3.6)	5.6 (4.6)	0.65
**UAC ratio mg/g (SD)**	152.3 (257)	88.6 (83.0)	123.7(196.9)	0.45
**Socioeconomic class**				0.98
**High**	1	1	2	
**Middle**	6	5	11	
**Low**	4	3	7	

**Duration of diabetes and Microalbuminuria:** stratifying the study population into those with diabetes for less than 5 years and those with diabetes for 5 years and above, mean UAC ratio of 90.00 mg/g was lower in the former than the latter group 224.76mg/g but the difference was not significant, p = 0.476. For children with raised UAC (12), there were 8 who had diabetes for less than 5 years, and 4 for longer than 5 years. Though the mean UAC in those with diabetes for over 5 years was higher than the other group, the difference was not significant, 276.62mg/g vs 151.43mg/g, p value = 0.59. The difference was not significant after 2 months when these subjects´ UAC was repeated, p = 0.92, and the means were a lot closer together. The mean age for those who had diabetes < 5years was 12.41 years, and for those with diabetes > 5 years was 15.50 years, p value = 0.158.

**Figure 1:**
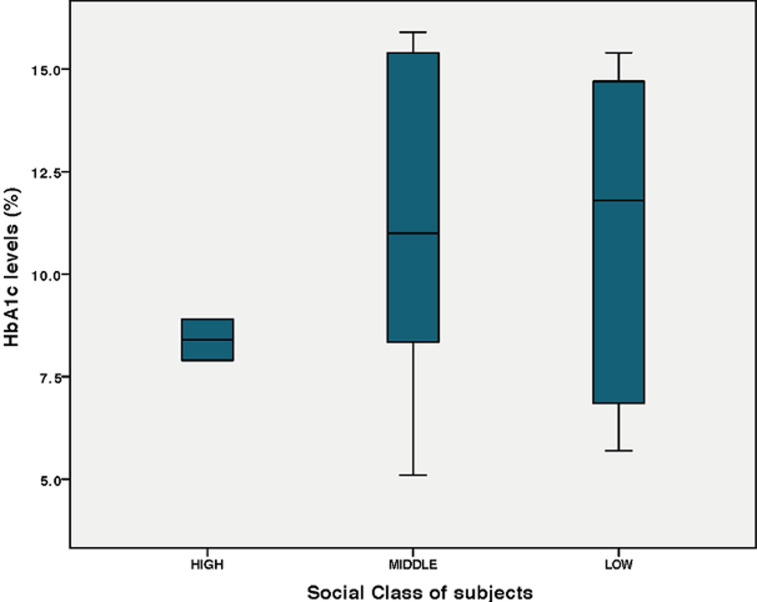
boxplot showing the mean HbA1c of children in high, middle and low social classes; note the stepwise increase in the mean HbA1c as the population moved from high to low social class

**Figure 2:**
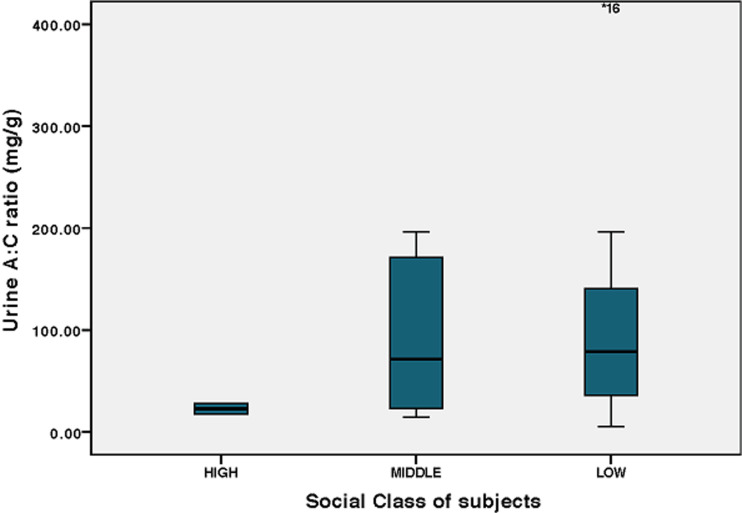
boxplot showing the mean UAC ratio across social classes with very low UAC ratio in the high social class and the outlier in the low social class group of very high urine AC ratio

**Insulin regimen and microalbuminuria:** there was no statistical difference in the mean UAC of those children who were on basal bolus regimen (88.68 ± 83.50) and those on mixtard (152.33 ± 257.09) p = 0.487, even though the mixtard group had higher mean values than their basal bolus counterpart. Repeat UAC done for children who had elevated UAC ratio was also high, 144.35 ± 127.00 mg/g though lower than the initial values 193.16 ±143.70 mg/g. **Social class and microalbuminuria:** glycaemic control was better in the high social class than the middle and low and this was same for the risk for renal impairment as none of the 2 children in the high social class had urine albumin creatinine ratio > 30 mg/g, as against the middle and low social classes (Figure 1, Figure 2).

## Discussion

The ISPAD and ADA guidelines recommend screening for microalbuminuria in childhood diabetes especially type 1 after 5 years of the disease process, or following 10 years of living with the disease, because microalbuminuria rarely occurs shortly after the disease is established [1]. However from our study, we notice some children developed microalbuminuria within 5 years of the disease process, which is similar to the report from Rwanda [6]. The prevalence of 60% in our study is higher than any other recorded study in, Africa [6] and the developed nations like Germany [10], USA, Sweden, though it must be stated that our population was small for generalisation. The study by Allyne *et al*. [5] published in 2010 had 23% for all samples with elevated urine albumin excretion, and of these, 31% were isolated and 9.3% persistent. Low incidence of microalbuminuria was also reported by Svensson *et al*. [11] in Sweden, though both studies had a higher cohort and their population was followed for more than 15 years unlike our cohort that is less than 10 years, where the possibility of status change can influence the glycaemic controls. Three studies in Africa reported high UAC ratios in children and all attributed these to poor glycaemic controls of the population though one was a systemic review [4, 12, 13].

Most of our children in Nigeria and our center depend on the insulin donated by Life for a child (LFAC) and these are mixtard insulin mostly with few regular insulin. The children have to wait a long time to get the donations and some go a long time without insulin. Some have even devised means of reducing their doses so the insulin vials do not finish before their next consignments arrive. Those on basal bolus regimen had better renal function as was demonstrated in the EDIC and many other studies. The children on basal bolus regimen had been on this for a year or less as they were changed to this regimen when they could afford this and due to their need for tighter glycaemic control. Continuing on this regimen could improve their glycaemic control and risk for renal damage. There was no age difference between those with elevated microalbuminuria and those with normal levels, possibly because most of the children in this cohort were already past the 10-year mark for screening for complications of diabetes. Two of the 3 children who were below 10 years had high UAC ratio level and repeat value were also high. All three also had high HbA1c levels and glucose values because it is difficult to control their feeding habits. Most of these children have a poor feeding habit and rarely adhere to dietary instructions because of fear of hypoglycaemia. HbA1c levels were high in the cohort as was in Tanzania. Though there was no correlation between HbA1c levels and UAC ratio, we believe this is because of the high proportion of children with poor control as was also seen in other African studies [4, 13, 14]. The study by Kalk *et al*. is quite interesting as it showed that African population in South Africa had higher UAC ratio than their white counterparts.

Poor glycaemic control is known to correlate with poor nutrition and insulin availability and adherence [1, 15]. The higher social class children had lower HbA1c compared to their low- and middle-class counterparts. This has been demonstrated in many studies and it relates to the availability of insulin, proper nutrition and adherence to dietary instructions with ability to check blood glucose levels more frequently [10, 16]. These children were also on basal bolus insulin regimen, which is known to help in tighter control of blood glucose [1, 11]. Spin off from this was the lower urine albumin creatinine ratio in children in high social class. Blood pressure in diabetes is a function of macrovascular and microvascular abnormalities and have been documented to be higher in poorly controlled diabetic children however all our patients had normal blood pressures, which was similar to the Rwanda cohort. Our study has shown a high prevalence of microalbuminuria which is related to poor glycaemic control, low social class, use of mixtard or free mix insulin and this can occur at any age and even within the 5 years of the disease process. We therefore recommend screening for risk of renal complications in children with type 1 diabetes who have poor glycaemic control irrespective of age or duration of disease in our population. Efforts should be made by all stakeholders to make basal bolus insulin regimen more affordable to all children with type 1 diabetes mellitus. The subject of justice in medical ethics flows through equality and /or inequality and distribution of resources [17]. Justice makes the argument that disparities within the social sphere should not be allowed in medical care but this is impossible in many resource-limited countries [18]. The national health insurance scheme has taken long term (chronic) diseases off their list so all patients pay out-of-pocket for insulin. Health allocations have been dismal in recent years in Nigeria never getting up to 2% the total budgetary allocation in the past 10 years [19]. Even then, most of the funds go to servicing recurrent expenditure, vaccines and malarial control as is seen in many other African and resource-limited countries [20]. While it is more expensive to use the basal bolus regimen, it is also the most efficient in tight glycaemic control, which must be maintained to prevent complications like we see in most of our patients.

Non-communicable diseases are less of priorities and arguable so in many resource-limited countries. Many more children will die from malaria, diarrhoea diseases or acute respiratory infections than the number that will have type 1 diabetes and who will eventually die from lack of insulin. However, the catastrophic health cost for families whose children have diabetes make it necessary for health care advocates to make insulin availability a priority so as not to drive these families into the impoverishing level because of the disease [21, 22]. Making insulin available used to be the priority for paediatric endocrinologists´ advocacy in Nigeria so they can manage their patients, but with improved care and survival rates, the goal has shifted to making regular and long acting insulin available for tighter glucose control and prevention of complications. While the endocrinologist will argue that resources be channeled to insulin for children, the health ministry and politicians will argue for the resources to go to primary health care with vaccine preventable diseases getting a larger proportion of the budget. The likelihood that a middle ground will be reached is foolhardy and farfetched, so a different approach must be used, which is special intervention funds and donor driven subsidized insulin for the most indigent of patients. This way, the drugs will sell at the cheapest possible price while cost of freighting and clearing from the ports will be borne by the donor agencies. The Federal government can also be made to give waivers for duty and clearing. While it is impossible to make everyone in the social sphere live in a certain class, the principles of justice in health care demand that basic amount of health care be provided to all citizens, and supplementing the care for those children with more expensive diseases, and from the low social classes [15]. All children with type 1 diabetes in need of basal bolus regimen should get this and every available resource must be channeled to make this possible except the family chooses otherwise. This limits the family´s moral burden of choosing continued care for their sick baby, or survival of other members of the family to prevent impoverishment. This study is limited by its small sample size and so a larger cohort study within the country or Africa or systematic review of the subject matter can be used for consensus statements if results are similar to our findings. Payment of healthcare out-of-pocket and lack of health insurance limit the number of important investigations that should be done for these sick children so researchers have to use their personal funds or seek grants for research purposes.

## Conclusion

The prevalence of microalbuminuria is high in our cohort of type 1 diabetes children and these were children on mixtard and had diabetes greater than 5 years. The children with type 1 diabetes mellitus who do not have regular access to insulin are likely to have earlier onset of long term renal complications showing as microalbuminuria. It is pertinent to ensure these children will not suffer further due to their lack and the health care system in Nigeria will find all ways possible to ensure regular delivery of insulin to these children.

### What is known about this topic

- Children with type 1 diabetes mellitus should be screened for renal and other long term complications after 5 years of disease onset or after age 10 years;
- Complications of diabetes mellitus are associated with poor glycaemic control.

### What this study adds

- Children with type 1 diabetes may have raised microalbuminuria even if the duration of diabetes is within 5 years;
- Elevated microalbuminuria and poor glycaemic control in this cohort is related to low socioeconomic class;
- Children on mixtard/free mix insulin regimen have raised microalbuminuria than those on basal bolus irrespective of duration of disease.
